# Poster 1008: Dialyzable leukocyte extracts as adjuvant treatment for allergic rhinitis

**DOI:** 10.1186/1939-4551-7-S1-P5

**Published:** 2014-02-03

**Authors:** Toni Angela Homberg, Rodolfo Ivan Lara, Sonia Mayra Pérez-Tapia, María Del Carmen Jiménez Martínez

**Affiliations:** 1 Instituto Politécnico Nacional, Mexico City, Mexico; 2 "Conde de Valenciana" Foundation, Mexico City, Mexico; 3 Universidad Nacional Autónoma de México, Faculty of Medicine, Mexico City, Mexico

## Background

Dialyzable leukocyte extracts (DLE) are human blood derived peptides less than 12kDa in size. DLE are hypothesized to induce a TH1 response, which makes them useful in the treatment of allergic disease. The purpose of this study was to determine the changes in symptomatology of patients with moderate and severe allergic rhinitis treated with standard treatment plus DLE.

## Methods

We analyzed the clinical files of 68 patients receiving dialyzable leukocyte extracts as adjuvant treatment for moderate to severe allergic rhinitis in the period between 2009 and 2013, at our immunology department. All patients receiving DLE signed an informed consent as part of a running protocol (IC-12-001) authorized by the ethics committee and health department. Patients had different standard treatments for allergic rhinitis, such as antihistamines, nasal corticosteroids, leukotriene inhibitors and immunotherapy in varying combinations. Oral DLE was added to standard treatment in patients that remained symptomatic. The initial DLE dose varied between 1 and 2 Units (5mL/Unit) per week.

## Results

28 (53.8%) female and 24 (46.2%) male patients ages 0-64 with a mean age of 17.3±17.5yrs with moderate to severe allergic rhinitis added DLE to their treatment. After 1 to 5 months, 52 (76.5%) reported improved symptoms, 11 (16.2%) abandoned treatment, 5 (7.4%) reported no improvement. No patients presented severe adverse effects. Of the 52 patients who reported improvement, 6 (11.5%) were asymptomatic, 32 (61.5%) mentioned general improvement of symptoms, 4 (7.7%) reported only decreased nasal congestion, 13 (25%) reported less frequent upper respiratory infections. 2 patients (3.8%) with concomitant asthma suspended the use of bronchodilators.

## Conclusions

DLE may be beneficial as adjuvant treatment for allergic rhinitis. Although patient self-reported improvement is subjective, this study lays the ground for more objective measurements of drug response in the future. Interestingly, it helped decrease the use of bronchodilators in 2 cases with concomitant asthma.

